# Understanding the Knowledge, Attitudes, and Practices of Young Obstetricians and Gynecologists (OBGYNs) in Menopause Management: Unveiling Insights From India

**DOI:** 10.7759/cureus.84444

**Published:** 2025-05-20

**Authors:** Jamuna Devi Gudidevuni, Apeksha Thakre, Ashish Bondia, Monika Bhanushali

**Affiliations:** 1 Obstetrics and Gynecology, Yashoda Hospitals, Secunderabad, IND; 2 Obstetrics and Gynecology, Cloudnine Hospital, Hyderabad, IND; 3 Medical Affairs, Pfizer Limited, Mumbai, IND

**Keywords:** attitude, estrogen, knowledge, menopausal hormone therapy, menopause, middle aged, postmenopausal, practice, public health, quality of life

## Abstract

Background

Menopause management poses a substantial public health challenge in India, characterized by a large and growing population of aging women. With more women undergoing this transition, there is growing interest in addressing its health implications. Despite its significance, there is a noticeable deficiency in the data concerning the knowledge, attitudes, and practices (KAP) among young Indian obstetricians and gynecologists (OBGYNs) regarding menopause management.

Objectives

As part of a pan-India investigation, this research aims to systematically assess the KAP related to menopause management among young OBGYNs by identifying the knowledge gap and evaluating the consistency of clinical practices with current guidelines.

Methodology

A comprehensive nationwide cross-sectional survey was conducted utilizing the CLIRNET digital platform, which facilitates collaboration and knowledge exchange among healthcare professionals in India. Our survey allowed open voluntary participation from 31,103 OBGYNs, of which 514 respondents met the inclusion criteria. Participants completed a detailed online questionnaire.

Results

The majority of respondents (474 of 514, 92.2%) correctly identified vasomotor symptoms as a primary complaint of menopause. Significant variations in knowledge and practice were observed based on years of experience. Among those with 0-3 years of practice, 54.1% accurately identified the average age of menopause in India, compared to 46.2% among those with 7-10 years of experience. In terms of management, 83.1% of OBGYNs with 0-3 years of experience recommended systemic estrogen combined with progestogen for vasomotor symptoms, whereas this figure dropped to 77.2% among the comparatively more experienced (7-10 years) cohort. Interestingly, younger OBGYNs were less likely to support the early initiation of menopausal hormone therapy (MHT), with only 30.4% in favor, compared to 48.9% among those with 7-10 years of experience.

Conclusion

The findings of this study reveal a marked heterogeneity in the knowledge base and clinical practices pertaining to menopause management among OBGYNs in India. This observed variability underscores the pressing need to develop and implement targeted, experience-sensitive educational interventions designed to address existing knowledge gaps and standardize the quality of menopause care across diverse clinical settings.

## Introduction

Menopause management is a public health challenge in India, driven by the substantial and growing cohort of women undergoing this physiological transition [1]. According to the 2011 Census of India, the total population was approximately 1.21 billion (121 crore), of which an estimated 96 million were women aged 45 years or older. In recent years, India's population has surpassed 1.44 billion, indicating a growing demographic of midlife and older women, whose health and well-being require focused attention [2]. This demographic is projected to surge to an estimated 401 million by 2026. The life expectancy for Indian women, currently at 70.3 years, is expected to rise to 77 years by 2050 [3]. These statistics underscore the imperative for effective strategies to manage menopausal symptoms as the population of postmenopausal women expands [4].

Ovarian senescence occurs gradually during the fourth and fifth decades of life, resulting in menopause and a changed hormonal milieu, with a prominent decrease in estrogen levels. The declining hormonal levels produce a variety of signs and symptoms, including hot flushes, profuse sweating, palpitations and tachycardia, vaginal dryness, and unstable mood [5,6]. The hypoestrogenic state accompanying menopause also correlates with increased risks of metabolic disorders, including type 2 diabetes mellitus (T2DM), cardiovascular diseases, and osteoporosis, collectively impairing the quality of life and increasing morbidity and mortality among postmenopausal women [7,8].

Research indicates that the average age of menopause in Indian women is 46.6 years, lower than in most other countries [9]. This earlier onset potentially leads to an increased prevalence of chronic conditions, such as osteoporotic fractures and T2DM, manifesting nearly a decade sooner than in their Caucasian counterparts as per the Indian Menopause Society guidelines [10,11]. A multicenter urban hospital-based study reported that 75% of postmenopausal Indian women experience vasomotor symptoms, which significantly impact daily life and overall well-being [11].

Menopausal hormone therapy (MHT) remains the gold standard for the management of menopausal symptoms, particularly vasomotor disturbances and chronic conditions associated with menopause [12]. MHT can enhance the vaginal health and quality of life in women experiencing menopausal symptoms, enhance bone density, and boost estrogen levels. It also maintains a favorable safety profile without significantly increasing adverse events or dyslipidemia risk [13]. However, the prescription of MHT by OBGYNs has declined due to controversies regarding its risk-benefit profile, particularly following the Women's Health Initiative (WHI) and the Million Women Study. These studies precipitated a sharp decline in MHT utilization, especially in Europe and the United States [14,15].

Professional organizations, including the Menopause Society and the Indian Menopause Society, recommend estrogen-progestogen therapy or estrogen therapy for the management of low bone mass and osteoporosis in early postmenopausal women with vasomotor symptoms, based on individualized risk-benefit assessments and regular evaluations. Understanding the knowledge, attitudes, and practices (KAP) of OBGYNs is essential for improving menopause management. Existing gaps in KAP, particularly in diagnosing menopause and managing its associated complications, underscore the need for targeted educational initiatives [11,16].

Research indicates that a significant proportion of women nearing menopause exhibit only moderate understanding of the condition, coupled with a pervasive apathy, which can be attributed to insufficient awareness [17]. Consequently, menopause management demands a sophisticated and multifaceted approach. The heterogeneity in the KAP among healthcare professionals, particularly within India's culturally diverse and densely populated context, highlights the critical need for extensive research. Such inquiry is essential to enhance menopause management protocols and to close the disparity between established guidelines and their implementation in clinical settings.

## Materials and methods

Study design

It was a cross-sectional, online survey conducted between April and May 2024 at CLIRNET, a digital interface promoting collaboration and exchange of knowledge connecting the public health domain in India. It provides a space for clinicians to engage in discussions, access medical insights, and stay updated on advancements in the field. The platform supports continuous learning and professional development within the medical community through its network of experts and digital resources. The online survey was distributed nationwide, covering Central India, East India, North India, Northeast India, South India, and Western India. Informed consent was obtained from each participant. The demographic data were well-distributed across three experience levels, ensuring a representative sample that accurately reflects the current landscape of comparatively young OBGYN practitioners.

The study utilized a voluntary, anonymized KAP survey conducted on an online doctor's platform, which, due to the absence of sensitive personal data, patient interaction, invasive procedures, and adherence to platform policies and user agreements, did not necessitate Institutional Ethics Committee (IEC) or Institutional Review Board (IRB) approval.

The questionnaire comprised 10 questions divided into three sections: Knowledge, Attitudes, and Practices (see Appendices). The questions addressed knowledge of MHT, attitudes toward menopause management strategies, and practices regarding MHT. These questions were based on current guidelines and expert consensus on menopause management.

Sample size estimation

Sample Size Calculation

To determine the necessary sample size for our finite population, we employed the following formula (for the sample size calculation for a finite population) [18]: $n=(Nz^2 p(1-p))/(e^2 (N-1)+z^2 p(1-p) )$. Here, n is the required sample size; N, population size; z, z-value (1.96 for 95% confidence level); p, estimated proportion of the population (0.5 was used for maximum variability); and e, margin of error (0.05 for 5%). Hence, the calculated required sample size is 380.

Sample Collection and Validation

The survey was conducted on the CLIRNET platform, utilizing its network of 31,103 registered OBGYNs. The voluntary response sampling was used, inviting any OBGYN visiting the platform to participate during the survey period. To maintain data integrity and reliability, the collected data was meticulously cleaned, eliminating any inconsistencies, inaccuracies, or incomplete entries. This process culminated in a final cohort of 514 OBGYNs who met the study requirements. This final cohort of 514 participants was used for detailed statistical analysis, ensuring the robustness and relevance of the findings. In designing the study, a high degree of precision and reliability was prioritized by setting a confidence level of 95% with a margin of error of 5%. In all cases, p<0.05 was considered statistically significant.

Statistical analysis

The KAP survey data were reviewed for completeness and accuracy. All responses were anonymized to ensure participant confidentiality. Demographic characteristics, including age, gender, and years of professional experience, were summarized using descriptive statistics. Means and standard deviations (SD) were calculated for continuous variables, while frequencies and percentages were used to describe categorical variables. These summaries provided a clear demographic profile of the sample and served as the foundation for subsequent analyses.

To examine associations between young OBGYNs' KAP regarding menopause management and their demographic characteristics, chi-squared tests were performed. Specifically, the relationships between years of professional experience and responses to the KAP questionnaire were assessed. A p-value of less than 0.05 was considered statistically significant. This analytical approach identified meaningful associations between respondents' experience and their reported practices and perceptions.

All statistical analyses were conducted using Microsoft Excel (2021, Microsoft Corporation, Redmond, Washington, United States) and R Version 4.3.3 (R Foundation for Statistical Computing, Vienna, Austria).

## Results

Demographic

The survey included a total of 514 OBGYNs. This demographic data provided a comprehensive representation of the target population, ensuring that the findings and insights gained from the research are relevant and applicable to professionals in this field. The inclusion of a substantial number of OBGYNs enhances the validity of the study's conclusions regarding menopause management and hormone replacement therapy. The respondents were predominantly young OBGYNs with an average of 5.34 years of professional experience. The demographic data were well-distributed across three experience levels, ensuring a representative sample that accurately reflects the current landscape of young OBGYN practitioners in India. This distribution facilitated a balanced analysis of how varying levels of professional experience influence attitudes and practices in menopause management (Table 1).

**Table 1:** Demographic profile and clinical experience of participating OBGYN respondents (n=514) OBGYN: obstetrics and gynecology

Variable	No. of respondents (n)	Percentage of respondents (%)	Mean±standard deviation
Level of experience			
0-3 years	148	28.8	5.34±2.71
4-6 years	182	35.4	
7-10 years	184	35.8	
Gender			
Female	436	84.8	-
Male	78	15.2	
Age	-	-	31.16±3.39

Knowledge

Our investigation evaluated the association between clinical experience and proficiency in menopause diagnostics, symptomatology recognition, and indications for MHT among young OBGYNs. A statistically significant correlation was observed between the duration of clinical practice and the accuracy of menopause diagnosis (p=0.046). Notably, diagnostic acumen, particularly in recognizing elevated follicle-stimulating hormone (FSH) levels and vasomotor symptoms, exhibited an improvement concomitant with increased experience, thereby emphasizing the imperative for enhanced educational strategies targeted at young practitioners.

Symptom recognition, specifically concerning vasomotor disturbances, vaginal atrophy, and mood fluctuations, demonstrated a statistically significant relationship with clinical experience (p=0.051). Although vasomotor symptoms were consistently identified across various experience levels (89.2-94%), other symptoms, such as fatigue and arthralgia, were less frequently recognized (Table 2).

**Table 2:** OBGYNs' responses to the knowledge questionnaire on menopause diagnosis, symptoms, and hormonal therapy, stratified by years of clinical experience (p<0.05 is considered statistically significant) OBGYNs: obstetricians and gynecologists; FSH: follicle-stimulating hormone; MHT: menopausal hormonal therapy

Questions	Total (n=514)		Experience (years)						P-value
			0-3 (n=148)		4-6 (n=182)		7-10 (n=184)		
	n	%	n	%	n	%	n	%	
The diagnosis of menopause requires which of the following:									
12 months of amenorrhea	186	36.19	68	45.95	59	32.42	59	32.07	0.046
An elevated FSH level	37	7.20	7	4.73	11	6.04	19	10.33	
Presence of vasomotor symptoms	15	2.92	5	3.38	4	2.20	6	3.26	
All the above	276	53.70	68	45.95	108	59.34	100	54.35	
Symptoms of menopause include (top 3):									
Vasomotor symptoms	474	92.22	132	89.19	169	92.86	173	94.02	0.051
Vaginal dryness	451	87.74	126	85.14	161	88.46	164	89.13	
Mood disturbance	356	69.26	95	64.19	129	70.88	132	71.74	
Fatigue	120	23.35	42	28.38	40	21.98	38	20.65	
Difficulty in memory/concentration	43	8.37	11	7.43	19	10.44	13	7.07	
Sleep disturbance	94	17.90	22	14.86	38	20.88	34	18.48	
Joint pain	56	10.70	15	10.14	22	12.09	19	10.33	
Weight loss	11	2.14	1	0.68	5	2.75	5	2.72	
The average age at natural menopause in India is:									
45	268	52.14	80	54.05	103	56.59	85	46.20	0.042
50	223	43.39	62	41.89	76	41.76	85	46.20	
56	21	4.09	5	3.38	3	1.65	13	7.07	
60	2	0.39	1	0.68	0	0.00	1	0.54	
I don't know	0	0.00	0	0.00	0	0.00	0	0.00	
Optimum starting time for MHT:									
Early menopause	208	40.47	45	30.41	73	40.11	90	48.91	0.015
Menopause within 5 years	250	48.64	85	57.43	87	47.80	78	42.39	
Menopause within 15 years	11	2.14	2	1.35	7	3.85	2	1.09	
Not clear	45	8.75	16	10.81	15	8.24	14	7.61	
Following are the indications of MHT:									
Menopausal symptoms	102	19.84	33	22.30	35	19.23	34	18.48	0.031
Urogenital atrophy	24	4.67	11	7.43	6	3.30	7	3.80	
Prevention of osteoporosis or fracture	67	13.04	30	20.27	20	10.99	17	9.24	
Primary ovarian insufficiency	49	9.53	11	7.43	17	9.34	21	11.41	
All the above	267	51.95	63	42.57	102	56.04	102	55.43	
Not clear	5	0.97	0	0.00	2	1.10	3	1.63	

Our study also revealed a significant variation in the awareness of the average menopausal age in India as a function of clinical experience (p=0.042). Among younger OBGYNs, 54.1% accurately identified the mean menopausal age as 45 years, compared to 46.2% among practitioners with 7-10 years of experience.

Furthermore, experience significantly impacted the understanding of optimal MHT initiation timing (p=0.015), with comparatively more years of experience, OBGYNs more frequently advocating for early intervention in alignment with the North American Menopause Society (NAMS) (now known as The Menopause Society) guidelines. The outcomes of this study emphasized the essential role of clinical experience in enhancing menopause management competency while simultaneously highlighting the necessity for ongoing education across all levels of practice to ensure optimal care for menopausal women. 

Attitudes

Our study evaluated the attitudes of young OBGYNs in identifying contraindications to MHT and assessed their perspectives on managing menopausal symptoms, uncovering significant correlations. History of venous thromboembolism was identified by 41.9% of OBGYNs with 0-3 years of experience and 26.6% with 7-10 years of experience; 35.4% of total respondents identified it as a contraindication. Undiagnosed genital bleeding was identified by almost 37% of respondents. The recognition of contraindications such as a history of breast cancer, porphyria, and hypertension remained consistently suboptimal across all experience groups. 

Attitudes toward menopausal management exhibited considerable variation; although a majority endorsed an individualized approach, a significant subset perceived menopausal symptoms as a natural aspect of aging, with approximately 25% advocating for proactive management strategies. There is a common thread of recognizing the importance of individualized treatment among OBGYNs, but there are also significant differences in attitudes toward treating menopausal symptoms as a natural part of aging or advocating for active management. These differences underscored the diversity of professional opinions shaped by varying experience levels and possibly differing educational backgrounds or clinical exposures. This data highlighted the importance of continued education within the OBGYN community to align on best practices for managing menopausal symptoms, ensuring that patient care is individualized and evidence-based (Table 3).

**Table 3:** OBGYNs' responses on attitudes toward MHT, categorized by years of clinical experience (p<0.05 is considered statistically significant) OBGYNs: obstetricians and gynecologists; MHT: menopausal hormonal therapy

Questions	Total (n=514)		Experience (years)						P-value
			0-3 (n=148)		4-6 (n=182)		7-10 (n=184)		
	n	%	n	%	n	%	n	%	
All but which of the following are contraindications to the use of MHT?									
Undiagnosed genital bleeding	190	36.96	49	33.11	60	32.97	81	44.02	0.009
Porphyria	35	6.81	9	6.08	18	9.89	8	4.35	
History of hypertension	37	7.20	6	4.05	15	8.24	16	8.70	
History of venous thromboembolism	182	35.41	62	41.89	71	39.01	49	26.63	
History of breast cancer	70	13.62	22	14.86	18	9.89	30	16.30	
Do you believe menopausal symptoms should be treated as a normal part of aging, or do you advocate for active management?									
Treat as a normal part of aging	163	31.71	51	34.46	47	25.82	65	35.33	0.209
Advocate for active management	126	24.51	35	23.65	44	24.18	47	25.54	
It depends on the individual cases	225	43.77	62	41.89	91	50.00	72	39.13	

Practices

The analysis of data represented in Table 4 revealed a significant consensus among respondents, with 81.71% advocating for the administration of systemic estrogen in combination with a progestogen for a 51-year-old female patient exhibiting severe vasomotor symptoms, with an intact uterus, and devoid of contraindications to MHT. This recommendation was consistent across varying levels of clinical experience, with 83.1% of practitioners having 0-3 years, 85.2% with 4-6 years, and 77.2% with 7-10 years of practice endorsing this therapeutic approach. This alignment reflects the robust evidence base supporting the efficacy of combined estrogen-progestogen therapy in mitigating vasomotor symptoms while protecting the endometrium from hyperplasia and carcinoma (Table 4).

**Table 4:** OBGYNs' responses to the practice questionnaire on the management of specific clinical case scenarios and the use of MHT, classified by years of clinical experience (p<0.05 is considered statistically significant) OBGYNs: obstetricians and gynecologists; MHT: menopausal hormone therapy

Questions	Total (n=514)		Experience (years)						P-value
			0-3 (n=148)		4-6 (n=182)		7-10 (n=184)		
	n	%	n	%	n	%	n	%	
For a 51-year-old woman with severe vasomotor symptoms who has a uterus and no contraindications to the use of MHT, which of the following would you recommend?									
Systemic estrogen only	49	9.53	15	10.14	14	7.69	20	10.87	0.269
Systemic estrogen plus a progestogen	420	81.71	123	83.11	155	85.16	142	77.17	
Progestogen only	45	8.75	10	6.76	13	7.14	22	11.96	
A 48-year-old woman, after hysterectomy, presents with severe vaginal dryness and itching and recurrent urinary tract infections. Which of the following would you recommend?									
Systemic estrogen only	19	3.70	5	3.38	3	1.65	11	5.98	0.499
Systemic estrogen plus a progestogen	18	3.50	5	3.38	7	3.85	6	3.26	
Low-dose vaginal estrogen	440	85.60	129	87.16	158	86.81	153	83.15	
Low-dose vaginal estrogen plus a progestogen	37	7.20	9	6.08	14	7.69	14	7.61	
Recommendation of MHT for premature ovarian insufficiency:									
For 3-5 years	78	15.18	33	22.30	21	11.54	24	13.04	0.12
At least until the mean age of menopause	284	55.25	79	53.38	99	54.40	106	57.61	
Until remission of symptoms	116	22.57	27	18.24	47	25.82	42	22.83	
Don't know	36	7.00	9	6.08	15	8.24	12	6.52	

In contrast, systemic estrogen monotherapy garnered significantly less support, with only 9.53% of respondents endorsing this option, indicative of the heightened risk for endometrial hyperplasia and malignancy associated with unopposed estrogen administration. Nonetheless, the selective application of estrogen monotherapy, particularly in scenarios where progestogen use is contraindicated, underscores the necessity for personalized treatment modalities in the management of menopause.

Furthermore, 85.6% of respondents strongly recommended low-dose vaginal estrogen therapy for post-hysterectomy patients experiencing severe urogenital symptoms, highlighting its effectiveness in addressing localized symptoms with minimal systemic absorption. The negligible preference for adjunctive progestogen therapy in this cohort reflects the absence of a uterus, eliminating the need for endometrial protection.

Finally, in the context of premature ovarian insufficiency, 55.25% of respondents advocated for the continuation of MHT until the average age of natural menopause, recognizing its critical role in reducing the long-term risks of osteoporosis and cardiovascular disease associated with premature ovarian insufficiency. These findings underscore the impact of clinical experience on MHT decision-making and emphasize the importance of ongoing education to enhance menopause management.

## Discussion

The outcomes from the KAP survey elucidate critical insights into the conceptualization and clinical execution of menopause management among Indian OBGYNs. Our findings echo global trends, such as those seen in China, where, in a study, though 98.3% of participants acknowledged the significance of menopause management, a substantial gap in comprehending MHT contraindications persists [19]. Concurrently, our study revealed that while young OBGYNs uniformly recognize the necessity of proactive menopause management, the depth of understanding, particularly regarding MHT contraindications, is predominantly observed among more experienced practitioners. The same trend was evident in the 2019 Mayo Clinic Proceedings, which highlighted significant deficiencies in menopause management knowledge among postgraduate medical residents [20]. These data from different geographical settings affirmed the variability in the comprehension of contraindications and diagnostic benchmarks for menopause.

Managing menopausal symptoms following a hysterectomy is an important finding from the KAP survey. Documented cases show that women who have a hysterectomy experience a sudden drop in estrogen, causing menopause symptoms like hot flashes, anxiety, depression, and reduced quality of life [20]. However, there is a frequent lack of comprehensive pre-surgical counseling about these potential complications, leading to fragmented care and unnecessary multi-specialist consultations. These findings emphasize the importance of structured follow-ups and targeted counseling, particularly regarding the timely initiation of MHT for women who undergo hysterectomy before reaching the typical age of natural menopause.

Continued training in hormone replacement therapy is essential for young OBGYN practitioners to effectively navigate the complexities of menopausal care, particularly in managing MHT and post-hysterectomy symptoms. This educational focus ensures that future OBGYNs are well-prepared to provide comprehensive care, reducing the risk of early menopausal symptoms and enhancing patient outcomes.

Additionally, our survey highlighted the importance of conducting FSH testing in patients presenting with hot flushes. This diagnostic measure can aid in accurately identifying menopausal transition and facilitate timely intervention, thus improving patient care and outcomes. The judicious use of FSH testing and a thorough clinical assessment represented a cornerstone of evidence-based menopause management.

It is also noteworthy that younger OBGYNs have shown a cautious approach when addressing premature ovarian insufficiency and its long-term treatment. With proper training and continuous education, these practitioners can be better equipped to provide long-term MHT treatment when required, ensuring patients receive the most effective and safe care over extended periods.

The management of menopausal symptoms involves a spectrum of treatment modalities, including the use of gynecological sprays, vaginal creams, paroxetine, and both oral and topical MHT. These options require meticulous consideration and individualized application. OBGYNs must actively enquire about menopausal symptoms in women who have reached menopause or are in the perimenopausal age group to identify and manage menopausal conditions effectively. Incorporating these practices into continuous medical education programs is crucial for ensuring high-quality care [12].

Moreover, our findings advocated for the development of demographic-specific educational initiatives tailored to the cultural and regional nuances of Indian women. The survey highlighted discrepancies among practitioners in identifying the average age of the natural onset of menopause in India. These regional variations necessitate the formulation of context-specific educational programs, ensuring that menopause management strategies are aligned with the unique demographic and cultural needs of the Indian population.

Establishing continuous professional development programs, endorsed by leading authorities in menopause research, will ensure that OBGYNs remain at the vanguard of emerging research and clinical guidelines. In turn, this will enhance the overall standard of care for menopausal women in India. Through these targeted interventions, we can effectively bridge the gap between current knowledge and clinical application, guaranteeing that all women receive the highest quality of care during this pivotal stage of life [21,22]. 

Notwithstanding the valuable insights generated, this study is subject to certain limitations that should be considered when interpreting the results. First, the use of self-reported data may have introduced response biases, including recall and social desirability bias, which can affect the validity of the responses. Second, the online nature of the survey could have resulted in selection bias, as participants with greater digital literacy or access to the internet may have been overrepresented. These methodological constraints may limit the extent to which the findings can be generalized to the wider population of OBGYNs, particularly those practicing in rural or resource-limited settings.

## Conclusions

This KAP survey highlights significant associations between clinical experience and various aspects of menopause management among young OBGYNs in India. Greater professional experience was significantly correlated with improved diagnostic accuracy, better recognition of menopause-related symptoms, and increased awareness of the appropriate timing for MHT initiation. While many viewed menopause as a natural aging process, there was a strong consensus on combined estrogen-progestogen therapy. However, variability in the usage of systemic estrogen monotherapy and MHT duration in premature ovarian insufficiency suggests a need for experience-sensitive educational strategies to address persistent knowledge and practice gaps, enhancing the quality of menopause care.

## Appendices

Knowledge, attitudes, and practices (KAP) survey questionnaire for healthcare professionals (HCPs)

Section 1: Knowledge

1. The diagnosis of menopause requires which of the following:

a. 12 months of amenorrhea

b. An elevated follicle-stimulating hormone (FSH) level

c. Presence of vasomotor symptoms

d. All the above

2. Symptoms of menopause include (top 3):

a. Vasomotor symptoms

b. Vaginal dryness

c. Mood disturbance

d. Fatigue

e. Difficulty in memory/concentration

f. Sleep disturbance

g. Joint pain

h. Weight loss

3. The average age at natural menopause in India is:

a. 45

b. 50

c. 56

d. 60

e. I don't know

4. Optimum starting time for menopausal hormonal therapy (MHT):

a. Early menopause

b. Menopause within 5 years

c. Menopause within 15 years

d. Not clear

5. Following are the indications of MHT:

a. Menopausal symptoms

b. Urogenital atrophy

c. Prevention of osteoporosis or fracture

d. Primary ovarian insufficiency

e. All the above

f. Not clear

Section 2: Attitudes

1. All but which of the following are contraindications to the use of MHT?

a. Undiagnosed genital bleeding

b. Porphyria

c. History of hypertension

d. History of venous thromboembolism

e. History of breast cancer

2. Do you believe menopausal symptoms should be treated as a normal part of aging, or do you advocate for active management?

a. Treat as a normal part of aging

b. Advocate for active management

c. It depends on the individual cases

Section 3: Practices

1. For a 51-year-old woman with severe vasomotor symptoms who has a uterus and no contraindications to the use of MHT, which of the following would you recommend?

a. Systemic estrogen only

b. Systemic estrogen plus a progestogen          

c. Progestogen only

2. A 48-year-old woman after hysterectomy presents with severe vaginal dryness and itching and recurrent urinary tract infections. Which of the following would you recommend?

a. Systemic estrogen only

b. Systemic estrogen plus a progestogen

c. Low-dose vaginal estrogen

d. Low-dose vaginal estrogen plus a progestogen

3. Recommendation of MHT for premature ovarian insufficiency:

a. For 3-5 years

b. At least until the mean age of menopause

c. Until remission of symptoms

d. Don't know
